# Evaluation of Aav Capsids and Delivery Approaches for Hereditary Hemorrhagic Telangiectasia Gene Therapy

**DOI:** 10.21203/rs.3.rs-4469011/v1

**Published:** 2024-06-12

**Authors:** Alka Yadav, Rich Liang, Kelly Press, Annika Schmidt, Zahra Shabani, Kun Leng, Calvin Wang, Abinav Sekhar, Joshua Shi, Garth W Devlin, Trevor J. Gonzalez, Aravind Asokan, Hua Su

**Affiliations:** University of California, San Francisco; University of California, San Francisco; University of California, San Francisco; University of California, San Francisco; University of California, San Francisco; University of California, San Francisco; University of California, San Francisco; University of California, San Francisco; University of California, San Francisco; Duke University School of Medicine; Duke University School of Medicine; Duke University School of Medicine; University of California, San Francisco

**Keywords:** Adeno-associated viral vector, hereditary hemorrhagic telangiectasia, brain arteriovenous malformation, Alk1, gene therapy

## Abstract

Nosebleeds and intracranial hemorrhage from brain arteriovenous malformations (bAVMs) are among the most devastating symptoms of patients with hereditary hemorrhagic telangiectasis (HHT). All available managements have limitations. We showed that intravenous delivery of soluble FMS-related tyrosine kinase 1 using an adeno-associated viral vector (AAV9-sFLT1) reduced bAVM severity of *endoglin* deficient mice. However, minor liver inflammation and growth arrest in young mice were observed. To identify AAV variants and delivery methods that can best transduce brain and nasal tissue with an optimal transduction profile, we compared 3 engineered AAV capsids (AAV.cc47, AAV.cc84 and AAV1RX) with AAV9. A single-stranded CBA promoter driven tdTomato transgene was packaged in these capsids and delivered intravenously (i.v.) or intranasally (i.n.) to wild-type mice. A CMV promoter driven *Alk1* transgene was packaged into AAV.cc84 and delivered to PdgfbiCre;*Alk1*^f/f^ mice through i.v. injection followed by brain AVM induction. Transduced cells in different organs, vessel density and abnormal vessels in the bAVMs, and liver inflammation were analyzed histologically. Liver and kidney function were measured enzymatically. Compared to other viral vectors, AAV.cc84, after i.v. delivery, transduced a high percentage of brain ECs and few hepatocytes; whereas after i.n. delivery, AAV.cc84 transduced ECs and perivascular cells in the brain, and ECs, epithelial cells, and skeletal muscles in the nose with minimum hepatocyte transduction. No changes to liver or kidney function were detected. Delivery of AAV.cc84-Alk1 through i.v. to PdgfbiCre;*Alk1*^f/f^ mice reduced bAVM severity. In summary, we propose that AAV.cc84-Alk1 is a promising candidate for developing gene therapy in HHT patients.

## Introduction

Hereditary Hemorrhagic Telangiectasia (HHT), also known as Osler-Weber-Rendu Syndrome, is an autosomal dominant disorder characterized by the development of telangiectasia (small arteriovenous malformations) on the mucosa and arteriovenous malformations (AVMs) in various organs including brain. HHT is the second most common genetic bleeding disorder affecting 1 in 5,000 individuals or approximately 60,000–70,000 Americans [1]. There is currently no FDA approved treatment for HHT patients [1].

Although several angiogenic proteins are dysregulated in HHT [2], elevated levels of vascular endothelial growth factor (VEGF) [3, 4] is of clinical interest due to the availability of targeted anti-VEGF agents. VEGF plays an important role in AVM pathophysiology [3, 5, 6]. VEGF stimulation is necessary for AVM formation and progression in the brain and skin of adult mice [5, 7–9]. Therefore, anti-VEGF could be a therapeutic option for HHT patients. However, systemic therapies with anti-VEGF agents, such as antibodies and kinase inhibitors, have many drawbacks [10–12] and need long-term repeated administration, because telangiectasias and bAVMs are chronic lesions, in which angiogenesis could be activated from time to time [13, 14].

Adeno-associated viral vector (AAV) mediated gene transfer has many advantages over direct systemic delivery of anti-angiogenic agents because it can mediate long-term transgene expression [15, 16], which makes repeated dosing unlikely to be needed. In addition, there are many engineered AAV capsids that make targeted delivery of therapeutic genes possible. We previously showed that in mice, intravenous (i.v.) delivery of AAV9-sFLT1, that has a recombinant AAV serotype 9 capsid and expressing the soluble FMS-related tyrosine kinase 1 (sFLT1), reversed bAVM phenotypes [17]. However, systemic sFLT1 expression caused minor liver inflammation and growth arrest of young mice [17]. Severe toxicity in nonhuman primates (NHPs) and piglets following high dose i.v. administration of AAV9-like vectors has also been reported [18, 19]. Thus, AAV9 is not an optimal vector for development of gene therapy for HHT patients.

To identify an AAV capsid(s) that can restrict therapeutic gene expression in the brain and nose with minimum hepatocyte transduction, we screened several engineered AAV capsids and discovered that one of the engineered AAV capsids, AAV.cc84, can predominantly transduce brain endothelial cells (ECs) after i.v delivery. More importantly, AAV.cc84 can transduce brain ECs and peri-vascular cells after intra-nasal (i.n.) delivery with minimal transduction of hepatocytes. Intra-nasal delivery of AAV.cc84 also transduced nasal epithelial cells and skeletal muscle cells.

The therapeutic efficacy of AAV.cc84 was tested in PdgfbiCre;*Alk1*^f/f^ mice that develop bAVMs upon induction of brain focal angiogenesis and *Alk1* deletion in ECs [8]. Delivery of AAV.cc84-Alk1 to PdgfbiCre;*Alk*1^f/f^ mice through i.v. mediated Alk1 expression in brain ECs, reduced vessel density and abnormal vessels, and increased vascular pericyte coverage in bAVMs. Therefore, AAV.cc84 is a promising vector for developing gene therapy strategy for treating HHT patients.

## Materials and Methods

### Engineered AAV capsids.

Three engineered AAV capsids (AAV.cc47, AAV.cc84 and AAV1RX) were produced using methods published in previous papers [20–23]. Their transduction efficiency and cell specificity were compared with AAV9. A single-stranded CBA promoter driven tdTomato transgene was packaged in these capsids to mediate ectopic expression of a red fluorescent protein (RFP). A CMV promoter driven human *Alk1* cDNA was packaged into AAV.cc84 to test the therapeutic efficacy.

#### Animals

All protocols and experimental procedures for using laboratory animals were approved by the Institutional Animal Care and Use Committee (IACUC) of the University of California, San Francisco (UCSF). Animal husbandry was provided by the staff of the IACUC, under the guidance of supervisors who are certified Animal Technologists, and by the staff of the Animal Core Facility. Veterinary care was provided by IACUC faculty members and veterinary residents located on the San Francisco General Hospital campus. All mice were housed in a pathogen-free area in 421×316 cm^2^ cages with 12 hours light/dark cycle.

Wild type (WT) C57BL/6 mice were purchased from the Jackson Laboratory (Bar harbor, ME) and used for testing viral transduction efficiency and specificity. *PdgfbiCreER;Alk*1^f/f^ mice that express an EC-specific TM inducible Cre recombinase and have their *Alk1* gene exons 4–6 floxed [24] were used to test the therapeutic efficacy of AAV.cc84-Alk1 vector.

#### BAVM model induction and viral vector delivery

For testing viral transduction efficiency and specificity, AAV vectors were i.v. or i.n. delivered to WT mice. Samples were collected 4 weeks after vector delivery. For i.v. delivery, 1×10^11^ viral genomes (vgs) were suspended in 100 ml of AAV formulation buffer combining 500 ml of 1X dPBS with 500 μl of 1 M MgCl2 and 50 μl of 10% (wt/vol) Pluronic F-68 [23] and were injected to each mouse through tail vein. For i.n. delivery, 20 μl of viral suspension containing 2×10^10^ vgs was dripped into the mouse nostrils, 10 μl each side.

For testing the therapeutic efficacy of AAV.cc84-Alk1, bAVMs were induced in *PdgfbiCreER;Alk1*^f/f^ mice through stereotactic injection of AAV-VEGF (2×10^9^ vgs) followed by intra peritoneal injection of tamoxifen (TM, 1.25 mg/25 g of mouse body weight) 14 days later[25]. AAV.cc84-Alk1 (1×10^11^ vgs) was delivered to mice through i.v. one day before bAVM model induction. Control mice were treated with AAV.cc84-RFP (1×10^11^ vgs). Samples were collected 10 days after TM treatment for analysis.

For injection of AAV-VEGF into the brain, mice were anesthetized by 4% isoflurane inhalation and were placed in a stereotactic frame with a holder (David Kopf Instruments, Tujunga, CA). A burr hole was drilled in the pericranium, 2 mm lateral to the sagittal suture and 1 mm posterior to the coronal suture. Two μl of viral suspension containing 2×10^9^ vgs of AAV-VEGF was stereotactically injected into the right basal ganglia, 3 mm underneath the brain surface, at a rate of 0.2 μl per minute using a Hamilton syringe. The needle was withdrawn after 10 minutes, and the wound was closed with a 4–0 suture.

### Immunofluorescence staining and image quantification.

For testing AAV transduction efficiency and specificity, mice were anesthetized with isoflurane inhalation and perfused with PBS through the left cardiac ventricle followed by 4% paraformaldehyde. The brains, hearts, lungs livers, intestines and noses were collected, incubated in 4% paraformaldehyde overnight, and then in 4% paraformaldehyde containing 30% sucrose for 1 week and then frozen in dry ice. Brains were sectioned into 20-μm-thick sections and the other organs were sectioned into 10-μm-thick sections using a Leica RM2155 microtome (Leica Microsystems, Wetzlar, Germany). RFP expression in the hearts, lungs and livers were directly observed on Vectashield (Cat #H-1500, Burlingame, CA,) mounted sections. RFP expression in the brains and noses were analyzed on sections co-stained with anti-RFP antibody (Cat # 632496, Takara Bio Inc., Japan) and anti-CD31 antibody (marker for ECs, AF3628, R&D Systems, Minneapolis, MN) under the Keyence microscope (model BZ-9000, Keyence Corporation of America, Itasca, IL). The distribution of RFP^+^ cells was quantified using NIH Image J 1.63 software. To analyze what cells were transduced in the brains, brain sections were also co-stained by anti-RFP antibody along with antibodies specific to neurons (NeuN, (1:500, Cat.# MAB377, Millipore, MA), vascular smooth muscle cells (a-SMA, 1:1000, Cat # A2547, Sigma-Aldrich, St. Louis, MO), pericytes (NG2, 1:50, Cat # MAB6689, R&D Systems, Minneapolis, MN), and astrocytes [glial fibrillar acidic protein (GFAP), 1:500, Cat # 13–0300, Calbiochem, La Jolla, CA].

To analyze the effect of AAV.cc84-Alk1 on bAVMs, the brain samples were frozen on dry ice and cut into 20-μm-thick coronal sections using a Leica CM1950 Cryostat (Leica Microsystems). Two sections per brain within the AAV-VEGF injection site were chosen for co-staining with antibodies specific to CD31 (1:100, Cat # SC-18916, Santa Cruz Biotechnology, CA) and Alk1 (1:50, Cat # AF770, R&D systems) or CD 31 and CD13 (marker for pericytes, 1:100, Cat # AF2335 R&D Systems) separately. Three images were taken from each section (right of, left of, and below the injection site) using the 20X objective lens of the Keyence microscope (model BZ-9000, Keyence Corporation of America). Vessel density and vascular pericyte coverage were quantified using NIH image J 1.63 software. Dysplastic vessels (vessels with lumen size larger than 15 μm) were quantified manually. For all immunostaining, sections were incubated at 4°C overnight with the primary antibodies indicated above. Alexa Fluor 594-conjugated (1:500, Invitrogen, Carlsbad, CA) or Alexa Fluor 488-conjugated IgG (1:500, Invitrogen, Carlsbad, CA) were used as secondary antibodies.

#### Western Blot

To analyze the Alk1 protein expression, frozen cortical tissue was homogenized in 0.4 ml of RIPA buffer (Thermo-Fisher Scientific, catalog 89900) + protease and phosphatase inhibitors (Cat # 5871, Cell Signaling Technology, Danvers, MA) using a Power Gen 125 S1 tissue homogenizer (Product Code: FS-PG125, Fisher Scientific, Hampton, NH) at maximal RPM setting for 20 seconds. Protein concentration was determined using a microplate reader (Emax, Molecular Devices, Sunnyvale, CA) with BCA assay (Cat # A55865, Thermo-Fisher Scientific) and then balanced across samples using RIPA + protease/phosphatase inhibitors as the diluent. Laemmli buffer (2x, Cat # 1610737, Bio-Rad, Hercules, CA) and 2.5% β-mercaptoethanol (v/v) were added, followed by heating at 70°C for 10 min, The samples were then stored at −80°C. For electrophoresis, 7.5 μg of protein was loaded to each lane of pre-cast 4–12% Tris-Glycine gels (Cat # NP0336B0X, Thermo-Fisher Scientific), Following electrophoresis, the protein was then transferred onto 0.2 μm nitrocellulose membranes (Cat # 1620146, Bio-Rad,) using Trans Blot Turbo (Bio-Rad) system. The membranes were air dried at 37°C for 20 min and then rehydrated in water prior to blocking. Membranes were blocked in Intercept blocking buffer (Cat # 927–60001, LI-COR, Lincoln, NE) for 30 min at room temperature and then incubated with rabbit anti Alk1 (1:500, Cat # ab263902, Abcam, Cambridge, MA; rabbit anti-beta actin (1:2,000, Cat # 4970, Cell Signaling Technology) in blocking buffer + 0.1% Tween-20 overnight at 4°C. After washing in Tris-buffered saline + 0.1% Tween-20 (TBST) 3 times (2 min each wash), the membranes were incubated with LI-COR Goat anti Rabbit antibody conjugated with IRDye800CW (Cat # 926–32211, LICOR Biotechnology, Lincoln, Nebraska); and Goat anti Rabbit antibody conjugated with HRP (Cat # 7074, Cell Signaling Technology); 1:20,000 in blocking buffer + 0.1% Tween-20 for 45 min at room temperature. Alk1 and beta actin bands were detected by Li-Cor Quantitative western blot scanner and quantified using Li-Cor imaging software.

#### Hematoxylin and eosin staining

Liver sections were stained with hematoxylin and eosin using a standard protocol. Ten sections from each liver were examined and imaged using a Keyence microscope to detect inflammatory cells.

#### Colorimetric assays for alkaline phosphatase activity, alanine transaminase activity and creatinine levels

Approximately 500 μl of blood was collected through the facial vein of each mouse, kept on ice for 30 minutes, and then centrifuged at 1,000 g for 10 minutes. Sera were collected for subsequent analyses. Alkaline phosphatase activity (ALP), alanine transaminase activity (ALT) and creatinine (Cr) levels in the sera were analyzed using colorimetric assays according to the manufacturer’s instructions (#ab83369, #ab105134 and #ab65340, respectively, Abcam, Cambridge, UK).

#### Transduction of human umbilical vein endothelial cells (HUVECs)

HUVEC cells (Cat. No. PCS-100–010^™^, ATCC Manassas, VA) were cultivated in Endothelium cell medium (Cat # 1001, Science cell, Carlsbad, CA) containing EC growth supplement (Cat # 354006, BD Biosciences, San Jose, CA) with 2% (v/v) fetal bovine serum (Cat # 26140–079, Thermo fisher Scientific), Penicillin/streptomycin solution (Cat.# 15140122, Thermo Fisher Scientific), F-12K Medium (Cat.# 21127–022, Thermo fisher Scientific), and heparin (Cat.# H3393, Sigma, St. Louis, MD), in a standard humidified incubator at 37°C with 5% (v/v) CO2. Passage 5 HUVECS were seeded in 4-well PCA chamber slides (Sarstedt, Nurnbrecht, Germany) at a cell density of 60,000 cells/well with 1 ml medium per well. Ten ul AAV.cc84-RFP virus (1×10^11^ vgs) were added to each well. Cells were washed with PBS 4 days later, fixed in 4% formalin for 20 min, and then mounted with Vectashield mounting medium containing 4,6-diamidino-2-phenylindole (DAPI, Vector Laboratories, Burlingame, CA). AAV.cc84 transduced cells were observed and imaged under the 20X objective lens of a Keyence microscope.

#### Statistical analyses

All quantifications were performed by at least two researchers who were blinded to the group assignment. Sample sizes for each experiment are shown in the figure legends. Data were analyzed using GraphPad Prism 9 software. Differences between two groups were analyzed using the unpaired two-tailed *t* test. Differences among multiple groups were analyzed by one-way ANOVA followed by multiple comparisons with Tukey’s correction. Data are presented as mean ± standard deviation (SD). A p value of less than 0.05 was considered statistically significant.

## Results

### AAV.cc84 transduces brain ECs predominantly after i.v. delivery

**Figure 1:**
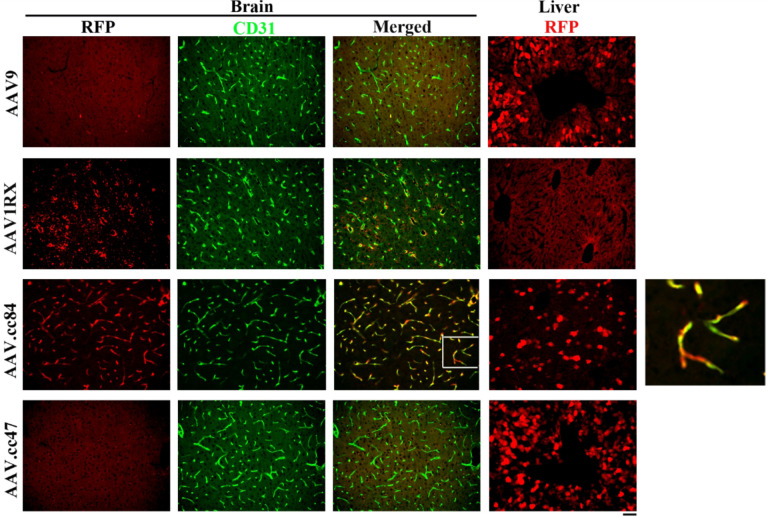
AAV.cc84 transduced brain ECs after i.v. injection with some hepatocyte transduction. AAV transduced cells were detected by their RFP expression (red, stained by an RFP specific antibody). Brain ECs were stained by an anti-CD31 antibody (green). The white square region in the AAV.cc84 merged picture is enlarged to show colocalization of RFP and CD31 staining. Scale bar: 50 mm.

We compared three engineered AAV capsids: 1RX, AAV.cc84 and AAV.cc47 with AAV9 vector in transducing normal brain cells in adult mice (8–12-month-old) through i.v. delivery. We found that AAV.cc84 transduced brain ECs predominantly. Similar to published data [17, 21, 22], AAV9 and AAV.cc47 transduced some brain cells and hepatocytes. AAV1RX transduced some brain ECs and other cells but not hepatocytes (Fig. 1).

AAV.cc84 transduced cells were spread evenly throughout the brain as determined by quantifying the ratio of RFP positive cells and brain ECs (CD31-positive) in four brain regions: bregma 4.4, 1.1, −1.7 and −5.8, (p = 0.84 by one-way ANOVA analysis, **Supplementary Fig. 1**).

AAV.cc47 transduced more hepatocytes than AAV9 (p = 0.001) and AAV.cc84 (p < 0.001, Fig. 1 **and Supplementary Fig. 2a**), an effect which was modified by gender. AAV.cc84 transduced significantly more hepatocytes in male mice than female mice (p < 0.001, **Supplementary Fig. 2b**). However, the brain EC transduction was similar between male and female (p = 0.67, **Supplementary Fig. 2c**).

AAV.cc84 also transduced a few neurons [0.57 to 8.33% of total neurons (NeuN^+^ nuclei) in the cortex and 3.5 to 11.11% in the basal ganglia]. In addition, AAV.cc84 transduced some pericytes, vascular smooth muscle cells, and astrocytes in the brain. AAV.cc84 also transduced cardiomyocytes and smooth muscle cells on the wall of intestines after i.v. delivery (**Supplementary Fig. 3**).

### AAV.cc84 transduced brain ECs and perivascular cells after i.n. delivery with minimal hepatocyte transduction

**Figure 2:**
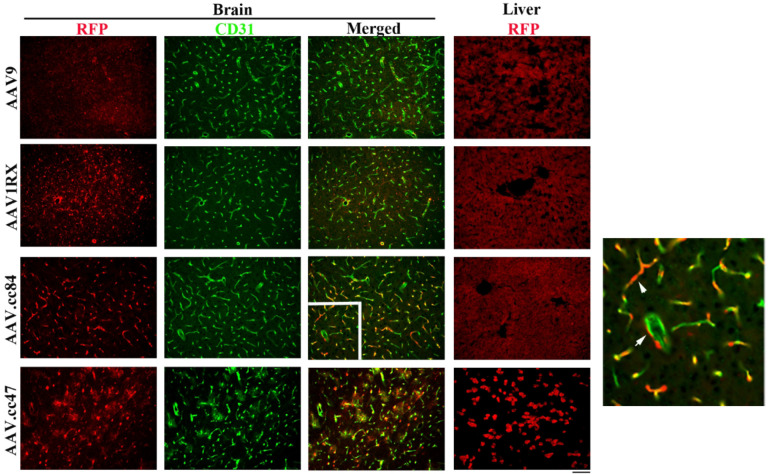
AAV.cc84 transduced brain ECs and perivascular cells after i.n. delivery. Transduced cells were detected by their RFP expression (red, stained by an RFP specific antibody). Brain ECs were stained by an anti-CD31 antibody (green). The white square region in the AAV.cc84 merged picture is enlarged to show colocalization of RFP and CD31 staining (Arrow head) and RFP^+^ perivascular cells (Arrow). Scale bar: 50 mm.

To test if i.n. delivery of the vectors tested above can minimize liver transduction, 10 ul of each vector (1×10^10^ vgs) was delivered to each nostril. We found that AAV.cc84 delivered through i.n. transduced both ECs and perivascular cells in the brain (Fig. 2). AAV.cc84 transduced more pericytes (50.5 ± 4.1% of FRP positive cells) after i.n. delivery than i.v. delivery (21.1 ± 5.8% of RFP positive cells, p < 0.001, **Supplementary Fig. 4b**). AAV9 transduced a few brain cells and AAV1RX transduced some ECs and other cells in the brain after i.n. delivery, a similar pattern as with i.v. delivery. Few hepatocytes were transduced by AAV.cc84, AAV9 and AAV1RX delivered i.n. Interestingly, AAV.cc47 transduced many brain cells and hepatocytes after i.n. delivery (Fig. 2). No RFP^+^ hepatocytes were detected in the livers of 8 male and 7 female mice after receiving AAV.cc84 i.n. The AAV.cc84 transduced cells were spread evenly throughout the brain after i.n. delivery, as assessed by quantifying the ratio of RFP positive cells and brain ECs (CD31 positive cells) in four brain regions: bregma 4.4, 1.1, −1.7 and − 5.8, (p = 0.84, analyzed by one-way ANOVA analysis, **Supplementary Fig. 5**).

In addition to ECs and pericytes, AAV.cc84 also transduced 0 to 2.43% neurons in the cortex and 1.38 to 3.96% of neurons in the basal ganglia, some astrocytes, vascular smooth muscles (**Supplementary Fig. 4**).

#### AAV.cc84 transduced nasal epithelial cells, ECs, and muscle tissue after i.n. delivery.

**Figure 3:**
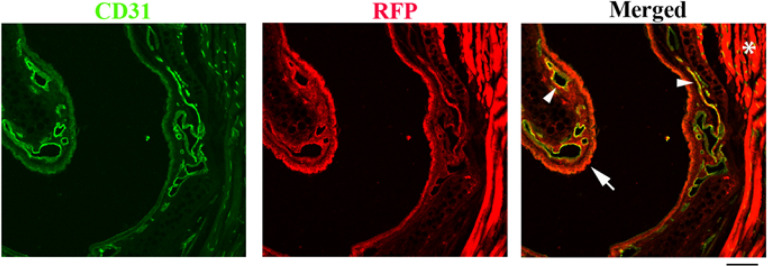
AAV.cc84 transduced nasal ECs, epithelial cells, and skeletal muscle cells after i.n. delivery. Transduced cells were detected by their RFP expression (red, stained by an RFP specific antibody). ECs were stained by an anti-CD31 antibody (green). * indicates skeletal muscles. Arrow indicated nasal epithelial cells and arrow heads indicate ECs. Scale bar: 100 mm.

Since epistaxis from nasal telangiectasias is one of the most devastating symptoms of HHT patients, to test if AAV.cc84 can be used to deliver therapeutic genes into the nose, we checked RFP expression in the nose after i.n. delivery of AAV.cc84-RFP. We found AAV.cc84 transduced nasal epithelial cells, ECs, and skeletal muscle cells (Fig. 3). Therefore, AAV.cc84 shows promise for treating nasal telangiectasias.

AAV.cc84 also transduced bronchial epithelial cells but not cardiomyocytes and hepatocytes after i.n delivery (**Supplementary Fig. 4**).

#### AAV.cc84 did not alter liver and kidney function.

To test if AAV.cc84 influenced liver and kidney function after i.v. or i.n. delivery, ALP, ALT, and Cr levels in the sera were analyzed. Their levels in AAV.cc84 treated mice were similar to the untreated controls for both i.v. and i.n. delivery (**Supplementary Fig. 6**).

#### Delivery of AAV.cc84-Alk1 i.v. reduced bAVM severity of mice with EC Alk1 deletion.

**Figure 4:**
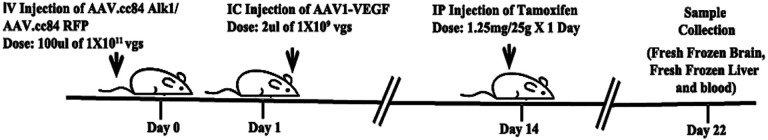
Design for AAV.cc84-Alk1 treatment. PdgfbiCre;*Alk1*^f/f^ mice were used. IV: intravenous injection; IC: intra-cranial brain injection; IP: intraperitoneal injection; vgs: viral genomes.

**Figure 5:**
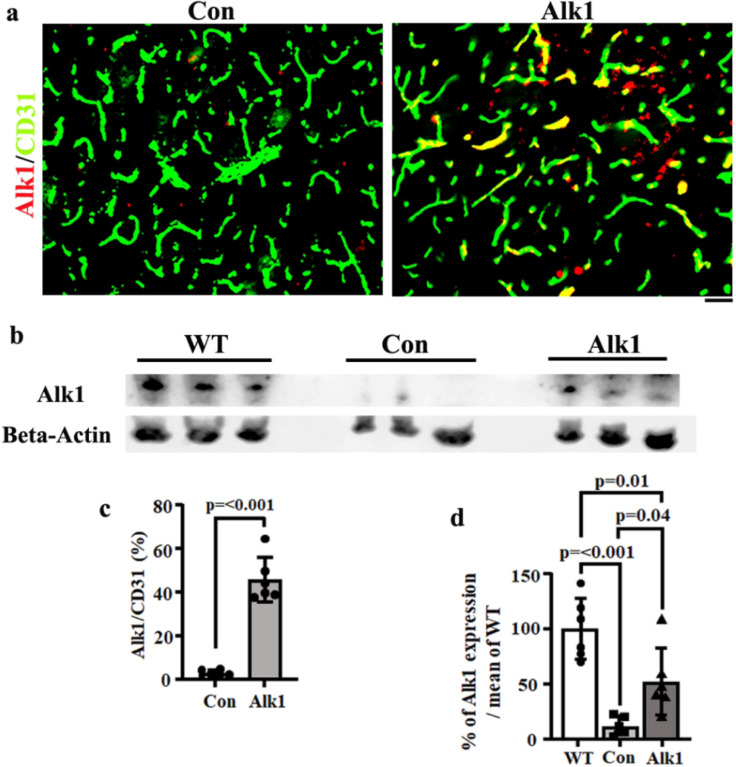
Intravenous injection of AAV.cc84-Alk1 mediated Alk1 expression in brain ECs and reduced the severity of bAVM phenotype. **(a)** Representative images of brain sections. ECs were stained by an anti-CD31 antibody (green), Alk1 were stained by an anti-Alk1 antibody (red). Scale bar: 50 mm. **(b)** Representative Western blot images. **(c)** Quantification of percentage of Alk1 positive ECs among total ECs**. (d)** Quantification of Alk1 protein expression as determined by western blotting (% of mean of WT Alk1 protein levels). WT: Samples from wild type mice; Con: AAV.cc84-RFP injected *Pdgfbi*CreER;*Alk1*^f/f^ mice; Alk1: AAV.cc84-Alk1 Injected *Pdgfbi*CreER;*Alk1*^f/f^ mice. n=6.

**Figure 6:**
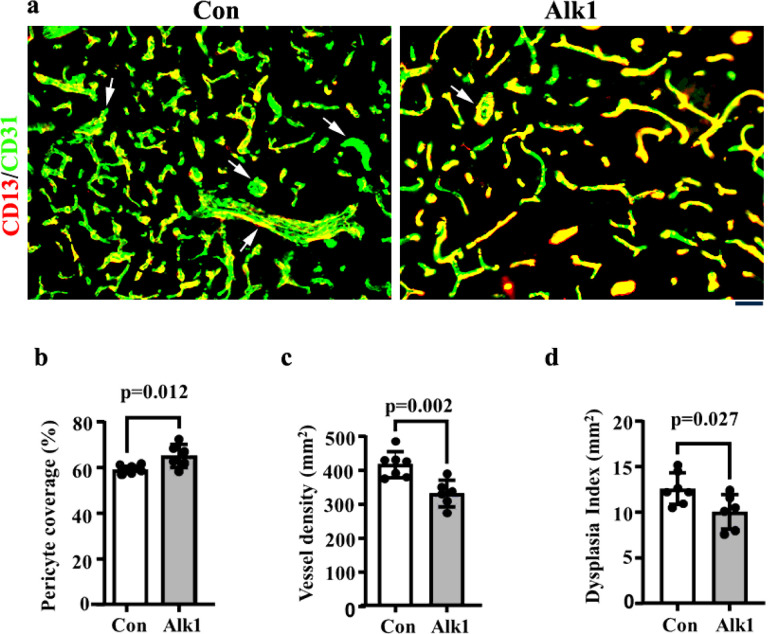
Delivery of AAV.cc84-Alk1 i.v. reduced the bAVM severity of *Pdgfbi*CreER;*Alk1*^f/f^ mice; **(a)** Representative images of brain sections. ECs were stained by an anti-CD31 antibody (green). Pericytes were stained by an anti-CD13 antibody (red). Scale bar: 50 mm. Quantification of vascular pericyte (CD13^+^) coverage (% of CD13^+^ and CD31^+^ cells verses CD31^+^ cells) **(b),** vascular density **(c)** and Dysplasia index (number of vessels with lumen size >15 mm/mm^2^) **(d).** Arrows indicate abnormal vessels; Con: AAV.cc84 RFP injected *Pdgfbi*CreER;*Alk1*^f/f^ mice, Alk1: AAV.cc84-Alk1 Injected *Pdgfbi*CreER;*Alk1*^f/f^ mice. n=6–7.

To test the potential use of AAV.cc84 in treating HHT bAVMs, we delivered AAV.cc84-Alk1 through i.v. injection to *Pdgfbi*CreER;*Alk1*^f/f^ mice one day before the induction of bAVMs. Brain samples were collected 22 days later (Fig. 4). Western blot analysis showed that TM treatment abolished about 89% of Alk1 expression in the brain compared to AAV.cc84-RFP treated controls (p < 0.013). Mice that received AAV.cc84-Alk1 through i.v. injection had a higher level of Alk1 protein in the brain (51.8 ± 30.5% of mean level in WT brain) than AAV.cc84-RFP treated mice (11.2± 8.8% of the mean level in WT brain, p = 0.04) Figs. 5b & 5d). Immunofluorescence staining showed more Alk1^+^ ECs in the bAVMs of *PdgfbiCreER;Alk1*^*f/f*^ mice in AAV.cc84-Alk1 treated group (45.8 ±10.2% of total ECs) than AAV.cc84-RFP treated control group (2.8 ± 1.4%, Figs. 5a & 5c). AAV.cc84-Alk1 treatment reduced vessel density (RFP treated control mice: 413.8± 38.26/mm^2^ vs Alk1 treated mice: 328.7± 38.96/mm^2^, p = 0.002) and dysplastic vessels (RFP treated controls: 12.60± 1.711/mm^2^ vs Alk1 treated mice: 10.03± 1.923/mm^2^, p = 0.027). AAV.cc84-Alk1 treatment also increased vascular pericyte coverage (p = 0.012 compared to controls, Fig. 6). These data indicate that AAV.cc84 can be used to deliver therapeutic genes into brain ECs to treat bAVM.

### AAV.cc84 transduced cultured HUVECs

**Figure 7:**
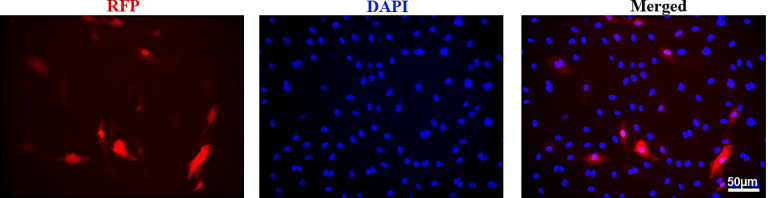
AAV.cc84 transduced human umbilical vein endothelial cells (HUVEC). Images were taken 4 days after addition of the virus into the culture. The nuclei were stained by DAPI. Scale bar: 50 μm.

To test if AAV.cc84 can transduce human ECs, we applied AAV.cc84-RFP to cultured HUBECs with 1:5×10^5^ vgs multiplicity of infection (MOI). RPF positive cells were detected 4 days after transduction (Fig. 7). Therefore, AAV.cc84 can be used to delivered therapeutic genes into human cells.

## Discussion

In this study, through screening 3 engineered AAV capsids, we found that AAV.cc84 is a promising vector for developing gene therapies to treat HHT bAVMs and nasal telangiectasia. AAV.cc84 was previously obtained by cross-species evolution in mouse and pig central nervous system following IV dosing[23]. Here, we demonstrate that AAV.cc84 transduces brain ECs predominantly after i.v. delivery and i.n. delivery. AAV.cc84 also transduced nasal ECs, epithelial cells, and skeletal muscle cells after i.n. delivery. In addition, AAV.cc84 can transduce cultured HUVECs, indicating that this vector can delivery genes into human ECs. Although AAV1RX could transduce brain ECs and other cells in the brain without transducing hepatocytes after i.v. and i.n. delivery, it was not as effective as AAV.cc84 and was very difficult to produce in large quantities. Although AAV.cc84 transduced some hepatocytes, cardiomyocytes, and smooth muscle cells on the intestine walls after i.v. delivery, minimal numbers of hepatocytes and cardiomyocytes were transduced after i.n. delivery.

Nasal bleeding and bAVM rupture are the major devastating symptoms of HHT patients. Ninety *%* of HHT patients develop recurrent, spontaneous epistaxis due to rupture of nasal telangiectasias, which have a profound impact on quality of life due to chronic bleed, development of iron deficiency anemia, and requirement of treatment with regular iron infusions and blood transfusions [26, 27]. Both surgical and pharmacological approaches are available for control of hemorrhage from mucosal telangiectasias, and these are often used in combination [28–31]. Anti-angiogenic approaches have the promise of reducing hemorrhage, modifying the disease process, and delaying development of telangiectasias in addition to potentially causing regression of existing vascular malformations [1]. However, these manipulations have limited efficiency and many limitations. Currently, there is no FDA approved treatment for prevent bleeding from nasal telangiectasia.

The rupture of bAVMs is unpredictable and life-threatening and can cause long-term disability. All current treatments for bAVM, including surgery, radiation, or endovascular embolization, are associated with considerable risks. Despite successfully achieving obliteration of the AVM nidus, there is growing recognition of AVM recurrence [32]. In addition, the treatment selection for patients with unruptured bAVMs has become increasingly controversial because the natural history for these patients may be less morbid than invasive therapies [33, 34]. As for other HHT phenotypes, no specific medical therapy is currently available to bAVM patients.

Mounting evidence shows that VEGF stimulation is necessary for AVM formation in the brain and skin of adult mice [5, 7–9]. Many agents can block VEGF signaling, e.g., anti-VEGF antibodies [1, 9, 35, 36] and tyrosine kinase inhibitors (TKIs) [37, 38]. However, these agents have considerable side effects [10, 11, 39]. In addition, TKIs showed a differential effect on skin and intestinal AVMs [40] and topical nasal spray of Bevacizumab (Avastin^®^), a recombinant, humanized VEGF antibody, did not improve epistaxis severity in the comparison to placebo treated patients [41, 42].

AAV-based gene therapy can mediate long-term transgene expression [15, 16], which makes repeated dosing unlikely to be needed. Previously, we showed in mice that i.v. injection of AAV9-sFLT1 reversed bAVM phenotypes [17]. However, uncontrolled systemic sFLT1 expression caused minor liver inflammation and growth arrest of young mice [17]. In this study, we identify an engineered AAV vector, AAV.cc84, that can limit sFLT1 expression in the brain and nose without significant hepatocyte transduction through i.n. delivery. Since sFLT1 is a secreted protein, all transduced cells can release sFLT1 to its surrounding tissue. Cell-specific transduction is not crucial. The reduced hepatocyte transduction is key to reducing systemic side effects.

Mutant HHT causative genes in ECs is essential for AVM initiation. Naturally, correction of Eng or Alk1 gene expression can rescue AVM phenotype [43]. Kim et al. reported that overexpression of Alk1 in ECs can rescue phenotype in mice with *Alk1* or *Eng* knocked out in ECs, without untoward effects [44]. This observation indicates that ECs of all subtypes can tolerate ectopic expression of Alk1. Here, we showed that AAV.cc84 can transduce brain ECs after i.v. and i.n. delivery. We also showed in this study that i.v. delivery of AAV.cc84-Alk1 induced Alk1 expression in brain ECs and reduced bAVM phenotype severity in mice with *Alk1* deletion in ECs. Previously, we showed that the number of *Alk1* deficient ECs is positively correlated with bAVM severity [25]. In this study, although i.v. delivery of AAV.cc84-Alk1 restored Alk1 expression in only 45.8% brain ECs, it still reduced bAVM severity. Therefore, AAV.cc84 is a promising candidate for delivering an *Alk1* transgene into brain ECs and the nose of HHT subjects. The fact that overexpression of Alk1 rescued the phenotypes in *Eng* mutant mice suggests that overexpression Alk1 in ECs may be effective for both HHT2 (caused by *ALK1* mutation) and HHT1 (caused by *ENG* mutation) patients, which together make up more than 80% of the HHT patient population.

In summary, in this study, we have identified an engineered AAV capsid, AAV.cc84, that can transduce brain ECs after i.v. or i.n. delivery. This capsid also mediated transduction of nasal ECs, epithelial cells, and skeletal cells. More importantly, this vector can transduce cultured HUVECs. We have also demonstrated that AAV.cc84 mediated Alk1 overexpression in brain ECs rescued bAVM phenotypes in *Pdgfbi*CreER;*Alk1*^f/f^ mice. Therefore, this capsid can not only be used to mediated secreted protein expression but also can transduce therapeutic genes into brain ECs specifically.

## Data Availability

The authors declare that all data supporting the findings of this study are available in the paper and its Supplementary Information.
